# Exploring physiological factors underlying individual differences in exercise‐induced blood pressure responses

**DOI:** 10.1113/EP093208

**Published:** 2025-11-26

**Authors:** Shigehiko Ogoh, Ryosuke Takeda, Narumi Kunimatsu, Hayato Tsukamoto, Ai Shimada, Tomoki Watada, Marina Feeley, Taichi Nishikawa, Marino Karaki, Kohei Watanabe, Tadayoshi Miyamoto

**Affiliations:** ^1^ Department of Biomedical Engineering Toyo University Asaka Saitama Japan; ^2^ Laboratory of Neuromuscular Biomechanics, School of Health and Sport Sciences Chukyo University Toyota Aichi Japan; ^3^ Faculty of Sport Sciences Waseda University Tokorozawa Saitama Japan; ^4^ Faculty of Sport and Health Sciences Osaka Sangyo University Daito Osaka Japan

**Keywords:** arterial stiffness, muscle metabolism, muscle oxidative capacity, postexercise muscle ischaemia

## Abstract

Even among healthy individuals, arterial blood pressure (ABP) responses to exercise vary widely. However, the mechanisms underlying this individual variability remain unclear. To investigate these mechanisms, 29 participants performed isometric handgrip exercise at 30% of their maximum voluntary contraction, followed by postexercise muscle ischaemia to assess metaboreflex activity. The systolic blood pressure response to exercise was not significantly correlated with arterial stiffness (carotid–femoral pulse wave velocity, *p* = 0.999, β = 0.000), peak oxygen uptake (*p* = 0.224, β = 0.168) or muscle oxidative capacity (*p* = 0.829, β = −0.049). In contrast, individual variability in systolic blood pressure was significantly associated with variability in heart rate during exercise (*p* = 0.013, β = 0.360) and the change in mean arterial pressure during postexercise muscle ischaemia (*p* = 0.014, β = 0.692). These findings suggest that peripheral characteristics are not primary determinants of individual differences in ABP responses to exercise in healthy young adults. Instead, variability in ABP responses might be more strongly influenced by individual differences in autonomic function. This pattern contrasts with the mechanisms underlying exaggerated ABP responses commonly observed in older adults and individuals with hypertension.

## Abstract (highlights)

- **What is the central question of this study？**
  There is considerable individual variation in blood pressure responses to exercise, even among healthy young individuals with normal resting blood pressure. Notably, an exaggerated blood pressure response has been linked to a higher risk of cardiovascular disease, yet the physiological mechanisms underlying this variability remain unclear.
- **What is the main finding and its importance？**
  Our findings suggest that central autonomic mechanisms, rather than peripheral cardiovascular factors, such as arterial stiffness, muscle oxygen capacity or muscle metabolism, might predominantly drive exaggerated blood pressure responses during exercise.

## 1 INTRODUCTION

Chronic exercise reduces cardiovascular disease (CVD) risk (Jabbarzadeh Ganjeh et al., 2024), and isometric training has been shown to lower resting arterial blood pressure (ABP) (Yamada et al., 2022). However, even brief bouts of isometric exercise can transiently elevate ABP, especially in older adults (Fisher et al., 2008) and those with CVD (Mizuno et al., 2013), making high‐intensity exercise potentially risky (Ainslie et al., 2007; Mundal et al., 1994; Schultz et al., 2015). Given that controlled hypertension carries a similar risk of mortality to normotension (Zhou et al., 2018), low‐ to moderate‐intensity exercise is recommended to prevent CVD (Kokkinos & Papademetriou, 2000). Interestingly, even in young, healthy individuals, ABP responses to exercise vary widely (Kunimatsu et al., 2024; Lauer et al., 1992; Mundal et al., 1994; Schultz et al., 2015), and some exhibit exaggerated increases despite normal resting ABP (Palatini, 1998; Schultz et al., 2015). Our recent studies (Kunimatsu et al., 2024; Washio & Ogoh, 2023; Washio et al., 2021) demonstrated that cognitive improvements are observed in individuals with low to moderate ABP responses, whereas individuals with higher ABP responses show diminished effects. This suggests that excessive ABP elevations during exercise might blunt cognitive gains, even in the absence of resting hypertension. These individual differences in ABP responses might critically influence the health benefits of exercise. Furthermore, an exaggerated ABP response to exercise in young, normotensive individuals has been associated with an increased risk of developing hypertension later in life (Odahara et al., 2010; Schultz et al., 2015; Zafrir et al., 2022). Thus, understanding the underlying mechanisms is important (even in young, healthy populations) for designing safe and effective exercise protocols. However, the physiological mechanisms behind such differences remain largely unclear.

Exercise‐induced ABP elevation is driven primarily by autonomic mechanisms, including central command and the exercise pressor reflex, which consists of mechano‐ and metaboreflex components (Ogoh et al., 2002; Raven et al., 2006). Variations in the relative contribution of these components might account for individual differences in ABP responses. Other physiological factors might also play a role. Several studies (Haarala et al., 2020; Thanassoulis et al., 2012; Wuttichaipradit et al., 2024) suggest that arterial stiffness, assessed by the cardio‐ankle vascular index or pulse wave velocity (PWV), influences individual differences in exercise‐induced ABP responses. A cardio‐ankle vascular index value >8.0 might help to identify individuals at risk of hypertensive responses during exercise (Wuttichaipradit et al., 2024). Moreover, impaired microvascular and muscle metabolic function, particularly in individuals with low exercise capacity, has been associated with exaggerated ABP responses to exercise (Kokkinos et al., 2002). Dipla et al. (2017) further linked reduced muscle oxygen delivery and utilization, in addition to increased microvascular stiffness, to exaggerated ABP responses during exercise. Collectively, these findings suggest that peripheral vascular characteristics, such as arterial stiffness and muscle oxidative capacity or metabolism, might contribute to individual differences in ABP responses to exercise. This is physiologically plausible, because increased arterial stiffness and reduced muscle oxidative capacity might limit blood flow necessary to sustain muscle function, thereby enhancing the pressor response via the muscle metaboreflex. Supporting this, studies have shown that restricting blood flow (to simulate these conditions) increases ABP and activates the metaboreflex (Hori et al., 2021; Kaufman et al., 1983). However, most of these studies (Dipla et al., 2017; Kokkinos et al., 2002; Thanassoulis et al., 2012; Wuttichaipradit et al., 2024) examined older adults or patients with CVD, such as hypertension. In contrast, variations in arterial stiffness and muscle oxidative capacity or metabolism in young, healthy individuals are likely to differ from those observed in older adults and patients with CVD. Therefore, the mechanisms underlying ABP responses in this population might also differ. To date, however, the physiological basis of individual differences in exercise‐induced ABP responses among young, healthy individuals remains unclear.

We hypothesized that, even in young, healthy individuals, differences in ABP response to exercise might be determined by arterial stiffness and muscle oxidative capacity or metabolism. Given that neural and cardiovascular functions improve with physical training, enhancing autonomic regulation and ABP control at rest and during exercise (Carter & Ray, 2015; Nystoriak & Bhatnagar, 2018), we also hypothesized that physical fitness contributes to individual variability in ABP response. To test these hypotheses, we examined the relationship between ABP response to handgrip exercise at 30% of maximal voluntary contraction (MVC) and several physiological factors: arterial stiffness [assessed by carotid–femoral PWV (cfPWV)], muscle oxidative capacity or metabolism [assessed using near‐infrared spectroscopy (NIRS), as previously described] and physical fitness [evaluated by maximal oxygen uptake (${\dot{V}}_{O_{2}\max}$)]. In addition, the heart rate (HR) response to handgrip exercise and ABP responses to post‐exercise muscle ischaemia (PEMI) were measured to evaluate the contributions of central command and the metaboreflex, respectively. The aim was to identify how each of these factors contributes to individual difference in ABP responses to exercise.

## 2 MATERIALS AND METHODS

### 2.1 Ethical approval

Twenty young men and nine young women participated in this study (mean age, 21 ± 1 years; height, 168.3 ± 8.7 cm; weight, 65.9 ± 11.6 kg). All participants were free of any known cerebrovascular, cardiovascular or pulmonary disorders and were not taking any medications at the time of enrolment. The participants exhibited a wide range of fitness levels, including individuals with no regular exercise habits, those who engaged in light to moderate exercise one to two times per week, and competitive athletes who trained daily. Prior to each experimental session, participants were instructed to refrain from consuming caffeinated beverages for 12 h and from engaging in strenuous exercise or consuming alcohol for 24 h. Each experiment was conducted ≥2 h after a light meal. The study protocol was approved by the Institutional Review Board of Osaka Sangyo University (approval no. 2024‐07). Written informed consent was obtained from all participants in accordance with the principles of the *Declaration of Helsinki*.

### 2.2 Experimental protocol

Upon arrival at the laboratory, participants were instrumented for HR monitoring using a lead II ECG (AT601G; Nihon Kohden, Tokyo, Japan) and for forearm muscle oxygenation measurement using a NIRS device (NIRO‐200NX, Hamamatsu Photonics, Hamamatsu, Japan). An automatic indirect manometer (EBP‐330, Minato Medical Science Co., Ltd, Osaka, Japan) was placed on the participant's left arm and recorded ABP, including systolic (SBP), diastolic (DBP) and mean ABP (MAP), at 30 s intervals. Each participant performed three MVC of handgrip (HG) exercise using the right arm to determine the exercise intensity (30% MVC). Following instrumentation, participants then rested in a supine position on a bed for ≥10 min prior to the start of the experimental protocol. Following the rest period, cfPWV and augmentation index (AIx) were assessed using a SphygomoCor XCEL system (AtCor Medical, Sydney, NSW, Australia). Baseline measurements for HG exercise were recorded during a 1 min quiet resting period. Participants then performed a 3 min isometric HG exercise at 30% MVC. Visual feedback was provided to help them maintain the target force output corresponding to the predetermined MVC percentage. Immediately before the end of the HG exercise, a cuff on the dominant arm was inflated to a suprasystolic pressure of 250 mmHg to induce PEMI by trapping forearm metabolites. After 3 min of occlusion, the cuff was deflated, and the participant was monitored for an additional 1 min. The HR and NIRS data were recorded continuously, whereas ABP was measured at 30 s intervals throughout the protocol. During the HG exercise, participants were instructed to avoid performing a Valsalva manoeuvre and remained in the supine position for the entire protocol. The 30% MVC intensity and 3 min duration were chosen based on previous studies showing that this workload increases MAP by ∼20%–30% (Washio et al., 2017). This intensity was considered sufficient to test our hypothesis while minimizing body movement and reducing the likelihood of Valsalva manoeuvres, which are more common at higher HG intensities or during other forms of exercises, such as leg squats (Perry et al., 2014).

### 2.3 Experimental measurements

Arterial stiffness was assessed by cfPWV and AIx, with AIx values corrected to a standard heart rate of 75 beats/min (AIx75). Additionally, participants were connected to an experimental apparatus to assess muscle oxidative capacity and metabolism using NIRS (Dipla et al., 2017, 2010). The NIRS device was placed on the forearm to monitor changes in muscle oxygenation non‐invasively by measuring micromolar (micromoles per litre) relative changes from baseline in oxygenated, deoxygenated and total haemoglobin levels. The tissue oxygen saturation index (TSI) was also recorded as an absolute measure of muscle oxidative capacity. Briefly, the NIRS protocol was performed after a 15 min rest period following probe placement, with participants lying supine on a hospital bed. The forearm was positioned at the heart level, and a pneumatic cuff was placed around the upper arm above the elbow. The cuff was rapidly inflated to a suprasystolic pressure of 250 mmHg to occlude blood flow to the forearm muscles and assess the maximal oxygen extraction capacity of skeletal muscles, which was verified by a plateau in total haemoglobin level indicating stable blood flow and volume. These procedures were performed ≥1 h before the HG exercise protocol.

### 2.4 Data analysis

#### 2.4.1 Arterial stiffness

Arterial stiffness was assessed using a vascular testing device [SphygomoCor XCEL Pulse Wave Velocity (PWV); AtCor Medical]. Pressure waveforms from the carotid and femoral arteries were collected sequentially to determine the foot‐to‐foot time interval (*T*_cf_), gated to the ECG. The value of *T*_cf_ was calculated as the time from the R‐wave to the foot (i.e., the start of the upstroke) of the femoral pulse wave (*T*_hf_), minus the time from the R‐wave to the foot of the carotid pulse wave (*T*_hc_). Carotid arterial pressure waveforms were measured using hand‐held applanation tonometry with a micro‐piezoresistive transducer placed over the left common carotid artery. Femoral arterial waveforms were recorded using a cuff placed on the left proximal thigh, connected to a plethysmographic sensor and an oscillometric pressure sensor. The body surface distance between the carotid artery and the suprasternal notch was measured with a tape measure and defined as the proximal distance (*L*_proximal_). The distal distance (*L*_distal_) was calculated as the sum of two segments: from the suprasternal notch to the umbilicus, and from the umbilicus to the top edge of the thigh cuff (Sugawara et al., 2021; Tomoto et al., 2023). The validity of cfPWV measurements using this thigh‐cuff method has been established (Butlin et al., 2013). Finally, cfPWV was calculated using the following equation: cfPWV = (*L*_distal_ − *L*_proximal_)/*T*_cf_. All measurements were performed three times with participants in the supine position. If the difference between repeated cfPWV values exceeded 0.5 m/s, the measurements were repeated to ensure consistency. We used the averaged data from three measurements as the cfPWV data.

#### 2.4.2 Muscle oxidative capacity

We also used NIRS to assess the TSI, as an absolute parameter for muscle oxygenation. The TSI was calculated as follows: TSI (%) = [O_2_Hb]/([O_2_Hb] + [HHb]) × 100, where [O_2_Hb] represents the concentration of oxygenated haemoglobin, and [HHb] represents the concentration of deoxygenated haemoglobin. The TSI occlusion slope and TSI reperfusion slope were calculated based on temporal changes in TSI during arterial occlusion and immediately after cuff release, respectively. The TSI occlusion slope and the magnitude of its decline were used as indices of muscle oxidative capacity. The TSI reperfusion slope and its magnitude were used as indices of the ability of vessels to accommodate the increased blood flow (microvascular reactivity) (McLay, Fontana et al., 2016; McLay, Nederveen et al., 2016). In addition, muscle oxygen consumption ($m{\dot{V}}_{O_{2}}$) was calculated based on the upsloping deoxygenated haemoglobin levels, assuming stable total haemoglobin (Gayda et al., 2015; Van Beekvelt et al., 2001). The $m{\dot{V}}_{O_{2}}$ was derived from NIRS using arterial occlusion by evaluating the rate of decreased in [Hb_diff_] ([O_2_Hb] − [HHb]), under the assumption that total haemoglobin concentration [tHb] ([O_2_Hb] + [HHb]) remains constant (De Blasi et al., 1997). The value of $m{\dot{V}}_{O_{2}}$ (in micromoles of O_2_ per litre per minute per 100 g) was calculated as follows: $m{\dot{V}}_{O_{2}}$ = |Δ([O_2_Hb] − [HHb])|/time (s) × 60 (convert to minutes)/10.4, where 10.4 represents the muscle density (100 g/L).

#### 2.4.3 Maximal oxygen uptake

The ${\dot{V}}_{O_{2}\max}$ was determined using a ramp incremental exercise test on a computer‐controlled bicycle ergometer (Fujin Raijin BU01, OCL Co., Tokyo). After a 3 min warm‐up at 20 W, the work rate was increased continuously by 1 W every 2 s (i.e. 30 W/min). Participants were instructed to maintain a pedalling cadence of ∼60 rpm throughout the test. The test was terminated when a participant was unable to maintain a cadence of >50 rpm despite strong verbal encouragement. The ${\dot{V}}_{O_{2}\max}$ was defined as the highest O_2_ consumption value recorded during the test and was considered achieved if the following criteria were met: (1) a plateau in O_2_ consumption despite an increase in workload; and (2) a respiratory exchange ratio of ≥1.10. This procedure was performed separately from the HG exercise protocol, with ≥1 week interval between the two.

### 2.5 Statistical analysis

Baseline data during supine rest and ${\dot{V}}_{O_{2}\max}$ were expressed as the mean ± SD and range (Table 1). All other values were expressed as the mean ± SD after confirming normal distribution using Shapiro–Wilk tests. During the exercise protocol, haemodynamic values used for statistical analysis included the average measurement during the baseline period, the last measurement during the 3 min exercise period, and the last measurement during the 3 min PEMI period. The TSI variables were averaged for the baseline period, the last 30 s of the exercise period, and the last 30 s of the PEMI period. These variables were analysed using one‐way repeated‐measures ANOVA, followed by the Student–Newman–Keuls method as a *post hoc* test (Statistica 7.0; StatSoft). The relationship between baseline ABP and the changes in ABP response to HG exercise was evaluated using Pearson's correlation analysis. Forced entry multiple regression analysis was performed to assess the relationship between ABP response to HG exercise and haemodynamic variables, $m{\dot{V}}_{O_{2}}$, arterial stiffness factors, ${\dot{V}}_{O_{2}\max}$ and absolute HG force. A *p*‐value of <0.05 was considered statistically significant.

**TABLE 1:** Average of haemodynamic, arterial stiffness and muscle oxygenation parameters during supine rest, and physical fitness and handgrip strength.

Type of parameter	Parameter	Units	Average ± SD	Range
Haemodynmaic parameters	HR	(beats/min)	63 ± 9	45–81
Arterial stiffness parameters	AIx		10.2 ± 9.5	−13.7 to 26.3
	AIx75		4.4 ± 11.2	−28.3 to 23.3
	cfPWV	(m/s)	6.2 ± 0.7	5.2–7.5
Muscle oxygenation parameters	$m{\dot{V}}_{O_{2}}$	(µmol/L/min/100 g)	0.792 ± 0.341	0.072–1.919
	TSI slope (occlusion)		−0.13 ± 0.06	−0.38 to 0.00
	TSI slope (reperfusion)		1.94 ± 1.17	0.18–4.56
Physical fitness	${\dot{V}}_{O_{2}\max}$	(mL/min/kg)	37.7 ± 9.5	24.8–65.6
Handgrip exercise	MVC	(N)	448.1 ± 140.0	93.5–735.0
	30% MVC absolute force	(N)	123.6 ± 39.5	27.8–201.7
	30% MVC relative force	(%)	27.7 ± 2.3	17.8–29.7

*Note*: Values are presented as the mean ± SD and range (*n* = 28). Abbreviations: AIx, augmentation index; AIx75, AIx corrected for a mean heart rate of 75 beats/min; cfPWV, carotid–femoral pulse wave velocity; HR, heart rate; MVC, maximal voluntary contraction; $m{\dot{V}}_{O_{2}}$, muscle oxygen consumption; TSI slope occlusion, change ratio (slope) of tissue oxygen saturation index during occlusion from baseline per time; TSI slope reperfusion, change ratio (slope) of tissue oxygen saturation index during reperfusion (cuff release) from occlusion over time; ${\dot{V}}_{O_{2}\max}$, maximal oxygen uptake.

## 3 RESULTS

**FIGURE 1:**
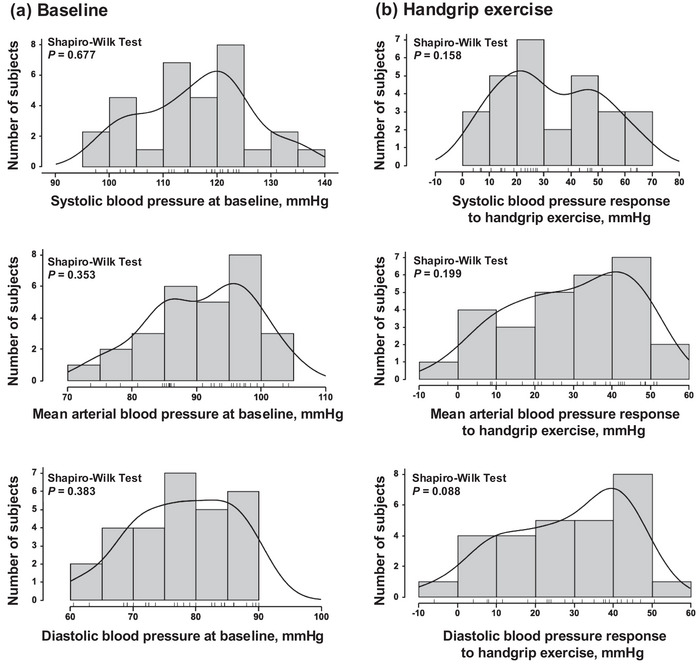
Distribution of systolic (upper), diastolic (lower) and mean blood pressure (centre) at baseline (a) and changes in blood pressure responses to handgrip exercise (b). A curved line indicates the density of the histogram (*n* = 28).

**FIGURE 2:**
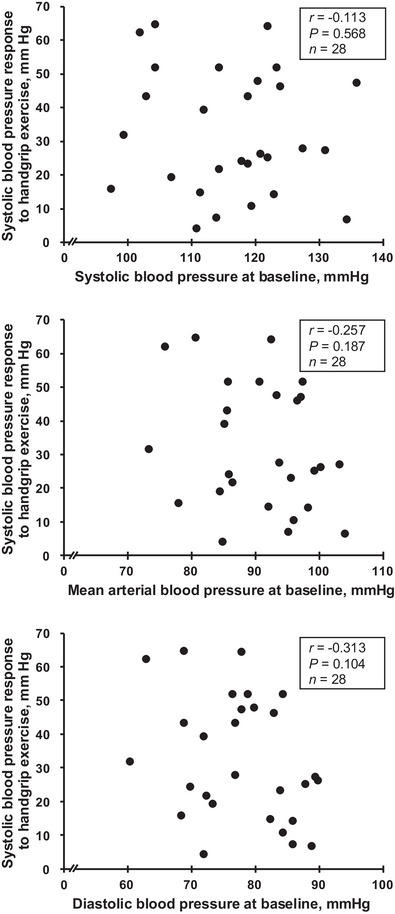
Regression analysis between systolic (upper), diastolic (lower) and mean blood pressure (centre) at baseline and changes in systolic blood pressure responses to handgrip exercise.

One subject had high blood pressure (DBP >90 mmHg) at rest in the recordings and was excluded, hence data from 28 healthy subjects were used in the analysis. Except for this subject, the young participants in this study were healthy and exhibited normal haemodynamic parameters, including HR and ABP, with all values falling within the normal range for healthy individuals (Table 1; Figure 1). The individual variability in resting ABP followed a normal distribution, as confirmed by the Shapiro–Wilk test (SBP, *p* = 0.677; MAP, *p* = 0.353; DBP, *p* = 0.383; Figure 1). The HG exercise significantly increased HR and ABP (SBP, DBP and MAP, all *p* < 0.001; Table 2), while TSI decreased significantly (*p* < 0.001), although pulse pressure remained unchanged (*p* = 0.070; Table 2). Importantly, the SBP, MAP and DBP responses to exercise followed a normal distribution, as confirmed by the Shapiro–Wilk test (SBP, *p* = 0.158; MAP, *p* = 0.199; DBP, *p* = 0.088; Figure 1) but exhibited a wide range: +4 to +65 mmHg for SBP, −3 to +52 mmHg for MAP, and −6 to +51 mmHg for DBP. Furthermore, the large variability in SBP responses was not associated with resting ABP (Figure 2). During the PEMI phase, HR returned to baseline levels, whereas SBP, MAP and DBP remained significantly elevated compared to baseline (all *p* < 0.001), again with a wide range: −5 to +43 mmHg for SBP, −4 to +38 mmHg for MAP, and −8 to +35 mmHg for DBP. In the multiple correlation analysis, the SBP response to exercise was significantly associated with both the HR response to exercise (β = 0.360, *p* = 0.013) and the MAP response to PEMI (β = 0.692, *p* = 0.014; Table 3). However, the SBP response to exercise was not significantly associated with cfPWV (β = 0.000, *p* = 0.999), $m{\dot{V}}_{O_{2}}$ (β = −0.049, *p* = 0.829), TSI slope occlusion (β = −0.064, *p* = 0.814), TSI slope reperfusion (*β* = −0.178, *p* = 0.408), ${\dot{V}}_{O_{2}\max}$ (β = 0.168, *p* = 0.224) and absolute HG force (β = 0.008, *p* = 0.977; Table 3). Similar patterns were observed in the associations between the MAP or DBP responses to exercise and those parameters: HR response during exercise, MAP response during PEMI, cfPWV, $m{\dot{V}}_{O_{2}}$, TSI slope, ${\dot{V}}_{O_{2}\max}$ or absolute HG force (Table 3).

**TABLE 2:** Haemodynamic and tissue oxygen saturation index response to handgrip exercise and postexercise muscle ischaemia.

Parameter	Units	Baseline	Exercise	PEMI	*p*‐Value
HR	(beats/min)	62 ± 9	87 ± 15*	64 ± 10^†^	<0.001
SBP	(mmHg)	116 ± 10	149 ± 20*	134 ± 15*, ^†^	<0.001
MAP	(mmHg)	91 ± 8	120 ± 14*	106 ± 12*, ^†^	<0.001
DBP	(mmHg)	78 ± 8	106 ± 13*	92 ± 11*, ^†^	<0.001
PP	(mmHg)	38 ± 8	43 ± 13	42 ± 10	0.070
TSI	(%)	70 ± 4	57 ± 12*	35 ± 13*, ^†^	<0.001

*Note*: Values are expressed as the mean ± SD (*n* = 28). Abbreviations: DBP, diastolic blood pressure; HR, heart rate; MAP, mean arterial pressure; PEMI, postexercise muscle ischaemia; PP, pulse pressure; SBP, systolic blood pressure; TSI, tissue oxygen saturation index.

* *P *< 0.05 vs. baseline.

^†^ *P *< 0.05 vs. Exercise.

**TABLE 3:** Multiple correlation analysis of blood pressure response to exercise with haemodynamic parameters, muscle oxidative capacity, arterial stiffness parameters, and physical fitness.

		*F*	Adjusted *R* ^2^	*P*	B	β
ΔSBP from baseline to Ex		7.019	0.684	<0.001	–	——
	ΔHR	–	–	0.013*	0.476	0.360
	ΔMAP PEMI	–	–	0.014*	1.088	0.692
	$m{\dot{V}}_{O_{2}}$	–	–	0.829	−2.559	−0.049
	TSI slope occlusion	–	–	0.814	−18.223	−0.064
	TSI slop reperfusion	–	–	0.408	−2.837	−0.178
	AIx	–	–	0.815	−0.096	−0.049
	cfPWV	–	–	0.999	0.009	0.000
	${\dot{V}}_{O_{2}\max}$	–	–	0.224	0.329	0.168
	HG force	–	–	0.977	0.003	0.008
ΔMAP from baseline to Ex		10.843	0.780	<0.001	–	–
	ΔHR	–	–	0.005*	0.389	0.351
	ΔMAP PEMI	–	–	0.006*	0.880	0.666
	$m{\dot{V}}_{O_{2}}$	–	–	0.985	−0.154	−0.003
	TSI slope occlusion	–	–	0.364	49.907	0.209
	TSI slop reperfusion	–	–	0.805	−0.586	−0.044
	AIx	–	–	0.237	−0.347	−0.211
	cfPWV	–	–	0.379	−3.470	−0.145
	${\dot{V}}_{O_{2}\max}$	–	–	0.421	0.151	0.091
	HG force	–	–	0.735	0.029	0.077
ΔDBP from baseline to Ex		8.347	0.726	<0.001	–	–
	ΔHR	–	–	0.016*	0.346	0.321
	ΔMAP PEMI	–	–	0.020*	0.776	0.604
	$m{\dot{V}}_{O_{2}}$	–	–	0.907	1.048	0.024
	TSI slope occlusion	–	–	0.167	83.973	0.362
	TSI slop reperfusion	–	–	0.835	0.539	0.041
	AIx	–	–	0.143	−0.473	−0.295
	cfPWV	–	–	0.229	−5.209	−0.224
	${\dot{V}}_{O_{2}\max}$	–	–	0.760	0.061	0.038
	HG force	–	–	0.653	0.042	0.114

Abbreviations: Alx, augmentation index; cfPWV, carotid‐femoral pulse wave velocity; HG, handgrip; HR, heart rate; MAP, mean arterial pressure; $m{\dot{V}}_{O_{2}}$, muscle oxygen consumption; PEMI, post‐exercise muscle ischemia; SBP, systolic blood pressure; TSI, tissue oxygen saturation index; ${\dot{V}}_{O_{2}\max}$, maximal oxygen uptake.

* *P* < 0.05 indicates a significant relationship.

## 4 DISCUSSION

Consistent with previous studies, the present study observed significant individual variation in the ABP response to exercise, characterized by a wide range of differences that followed a normal distribution (SBP, +4 to +65 mmHg; Figure 1). Exaggerated ABP responses are common in older adults and cardiovascular patients and are linked to arterial stiffness, reduced muscle oxidative capacity, and metabolism. However, such associations were not observed among young healthy participants. Therefore, the mechanisms behind ABP variability (e.g., exaggerated ABP response) in young healthy individuals are likely to differ from those in ageing or cardiovascular conditions.

It is known that some young, healthy individuals with normal resting ABP exhibit exaggerated ABP responses during exercise, similar to older adults (Fisher et al., 2008) and patients with CVD (Mizuno et al., 2013). This might indicate a future risk of hypertension, despite normal resting ABP (Odahara et al., 2010; Schultz & Sharman, 2014; Zafrir et al., 2022), although the underlying mechanisms remain unclear. Clarifying these mechanisms could support early prevention and optimize exercise benefits. The aim of this study was to address this important question. We hypothesized that young, healthy individuals who exhibit an exaggerated ABP response to exercise might share similar physiological characteristics with older adults and individuals with CVD. One such factor is muscle oxidative capacity, because its lower levels have been linked to higher ABP response to exercise in patients with hypertension (Dipla et al., 2017). In addition, we examined effects of muscle metabolism and physical fitness, given that reduced muscle oxidative capacity contributes to impaired muscle metabolism and decreased physical fitness. However, muscle oxidative capacity, metabolism and ${\dot{V}}_{O_{2}\max}$, as an index of physical fitness, were not associated with individual ABP responses to exercise (Table 3). We also investigated whether arterial stiffness contributes to the ABP response to exercise, because it is a known risk factor for hypertension (Kim, 2023) and might influence muscle oxidative capacity (Dipla et al., 2017). Contrary to our hypothesis, no significant relationship was found between arterial stiffness and muscle oxidative capacity (*r* = 0.056, *p* = 0.776), nor was there a significant association between cfPWV and ABP responses to exercise (Table 3). Notably, although cfPWV showed considerable interindividual variability, all values remained within the normal range (cfPWV, 5–7 m/s). Although a higher cfPWV has been associated with elevated resting ABP and exaggerated exercise‐induced ABP response in patients with CVD (Vasan et al., 2022) and older adults (Fisher et al., 2008), the impact of normal‐range arterial stiffness on ABP regulation in this population appears to be minimal. These findings suggest that the mechanisms underlying individual variation in the ABP response to exercise among young, healthy individuals differ from those observed in older adults and patients with cardiovascular diseases, such as hypertension.

The ABP during exercise is regulated by mechanisms such as central command and the exercise pressor reflex (mechanoreflex and metaboreflex), all mediated by the autonomic nervous system (Mitchell et al., 1989; Raven et al., 2006). Given that the mechanoreflex primarily influences ABP at the onset of exercise (Fadel, 2015), central command and the metaboreflex probably played a larger role than the mechanoreflex in the short‐duration exercise protocol used in the present study. Interestingly, significant correlations between ABP and HR responses to exercise (e.g., SBP, β = 0.360, *p* = 0.013) suggest that central command, which coordinates HR and cardiac output with exercise intensity, might contribute to individual ABP variability. However, afferent feedback from working muscles and sympathetic activation, especially during isometric contractions such as handgrip exercise, also influence cardiovascular responses. Therefore, individual differences in ABP response might result from variations in both central command and metaboreflex‐related sympathetic activation. Although arterial stiffness, muscle oxidative capacity and metabolism (factors linked to the metaboreflex) were not associated with this individual variability, strong correlations were observed between exercise‐induced ABP responses and those during PEMI (e.g. SBP, β = 0.692, *p* = 0.014), suggesting a major role of the metaboreflex in individual differences in ABP responses to exercise. This discrepancy implies that neural factors, rather than peripheral arterial or muscle properties, might underlie variations in the ABP response. Notably, the ABP response to PEMI reflects not only muscle metabolism but also neural mechanisms, including pathways from receptors to the brainstem (notably the nucleus tractus solitarii), signal processing within the nucleus tractus solitarii, and subsequent effects on sympathetic nerve activity and HR. Therefore, the findings of the present study suggest that neural factors related to the muscle metaboreflex contribute to the ABP response. Moreover, variations in exercise‐induced ABP responses, particularly exaggerated responses, might arise not from peripheral arterial or muscular properties but from sympathetic activation via central neural mechanisms, including cerebral pathways. However, we did not assess neural activity in the present study; therefore, future research using microneurography to measure muscle sympathetic nerve activity or neural blockade techniques is warranted.

A previous study by Greaney et al. (2015) also demonstrated that normotensive young individuals with a family history of hypertension exhibit a more pronounced ABP response to exercise in comparison to those without such a history. Moreover, individuals who experienced an exaggerated ABP response during childhood and adolescence (Alvarez‐Pitti et al., 2022) continue to exhibit this response into adulthood. Additionally, higher left ventricular mass in normotensive individuals has been associated with an exaggerated ABP response during exercise (Oh et al., 2018; Schultz et al., 2016), and increased left ventricular mass in childhood might serve as a significant predictor of future hypertension and its associated consequences (Mahoney et al., 1988). These findings suggest that genetic factors might contribute to individual differences in the ABP response during exercise. However, the mechanisms behind individual variations in ABP responses to exercise remain unclear. Further investigation is warranted to elucidate the contributory factors and identify the mechanisms responsible for individual variability in ABP responses to exercise.

### 4.1 Perspective and significance

A single bout of exercise temporarily elevates ABP to meet the metabolic demands of working muscles. In older adults and hypertensive patients, this response is more pronounced, and excessive rises can increase the risk of vascular damage. Interestingly, even young, healthy individuals show considerable variation in exercise‐induced ABP responses (Kunimatsu et al., 2024; Lauer et al., 1992; Mundal et al., 1994; Schultz et al., 2015), with some exhibiting exaggerated responses despite normal resting ABP (Palatini, 1998; Schultz et al., 2015), potentially increasing future risk of hypertension (Odahara et al., 2010; Schultz et al., 2015; Zafrir et al., 2022). Importantly, these individuals are often unaware that their ABP becomes excessively elevated during physical activity.

Previous studies define exaggerated blood pressure responses as a 10 mmHg rise in SBP per metabolic equivalent of task (MET) (Bond et al., 2012). For 30% MVC HG exercise (4–5 METs), this equates to ∼40–50 mmHg. However, these criteria are based on whole‐body exercise. Our prior work (Kunimatsu et al., 2024) suggests that an SBP increase > ∼45 mmHg during HG exercise predicts reduced cognitive benefits, providing a tentative threshold. Further research is needed to establish definitive criteria for small‐muscle exercises, although they are useful for screening.

Our findings suggest that variations in exercise‐induced ABP responses in young, healthy individuals are mainly attributable to central factors, such as autonomic regulation, highlighting the need for early identification and a comprehensive assessment beyond fitness or exercise habits.

### 4.2 Limitations

We acknowledge several limitations of this study. First, we used the ABP response to PEMI as an indirect indicator of metaboreflex activity. However, previous research (Ray & Mark, 1993) suggests that measuring muscle sympathetic nerve activity during PEMI would provide a more accurate assessment of metaboreflex function. Second, the unequal sex distribution among participants might have influenced our results. Given that both men and women were included and that menstrual cycles were not controlled in female participants, potential sex differences and hormonal fluctuations could have affected the outcomes. Future studies should account for these factors to clarify their influence on the observed results. Third, this study used forced entry multiple regression analysis to assess the relationship between ABP responses to HG exercise and nine independent variables. However, given the relatively small sample size, particularly in the context of multiple regression analysis, concerns arise regarding the generalizability of the findings to larger populations. To address this limitation, we also performed stepwise multiple regression and Pearson's/Spearman's rank correlation analyses, which yielded consistent results (data not shown to avoid duplication). Furthermore, a power analysis was conducted to estimate the sample sizes required to detect significant associations between individual differences in ABP responses to exercise and muscle oxidative capacity, arterial stiffness or physical fitness. The analysis indicated that sample sizes of *n* > 121, *n* > 1972 and *n* > 70, respectively, would be necessary to detect significant effects. Therefore, the absence of significant findings in the present study is unlikely to be attributed solely to insufficient statistical power. Nonetheless, future studies with larger cohorts are warranted to confirm these findings and enhance their generalizability.

## 5 CONCLUSION

Our results suggest that HG exercise increases blood pressure with substantial individual variation. Contrary to our hypothesis, this variation in ABP response was not associated with arterial stiffness, muscle oxidative capacity, muscle metabolism or physical fitness. However, individual differences in HR response to exercise and ABP response during PEMI were correlated with ABP responses, suggesting that variations in central command activation and muscle metaboreflex (particularly neural factors), rather than peripheral arterial or muscle properties, might underlie individual differences in the ABP response. Individuals with exaggerated exercise‐induced ABP responses might exhibit greater sympathetic activation via central neural mechanisms (i.e., cerebral pathways rather than peripheral ones). Nevertheless, further investigation is needed to confirm neural activity related to central command and the metaboreflex.

## AUTHOR CONTRIBUTIONS

Shigehiko Ogoh was responsible for the conception and design of the experiments. Shigehiko Ogoh, Ryosuke Takeda, Narumi Kunimatsu, Hayato Tsukamoto, Ai Shimada, Tomoki Watada, Marina Feeley, Taichi Nishikawa, Marino Karaki, Kohei Watanabe and Tadayoshi Miyamoto performed experiments. Shigehiko Ogoh and Ryosuke Takeda analysed the data. Shigehiko Ogoh drafted the paper. All authors edited the paper. All authors have read and approved the final version of this manuscript and agree to be accountable for all aspects of the work in ensuring that questions related to the accuracy or integrity of any part of the work are appropriately investigated and resolved. All persons designated as authors qualify for authorship, and all those who qualify for authorship are listed.

## CONFLICT OF INTEREST

The authors have nothing to report.

## Data Availability

All data supporting the results are presented in the manuscript.
